# The TEMPO integrator: accelerating molecular simulations by temporally multiscale force prediction

**DOI:** 10.1093/bioadv/vbaf142

**Published:** 2025-06-20

**Authors:** Reshef Mintz, Barak Raveh

**Affiliations:** School of Computer Science and Engineering, The Hebrew University of Jerusalem, Jerusalem 9190401, Israel; School of Computer Science and Engineering, The Hebrew University of Jerusalem, Jerusalem 9190401, Israel

## Abstract

**Motivation:**

Molecular dynamics (MD) simulations enable the study of complex biomolecular processes by integrating system forces over time, but their computational inefficiency limits application at relevant scales. Enhanced sampling methods often sacrifice kinetic detail and require prior knowledge of the energy landscape.

**Results:**

We developed the temporally multiscale prediction (TEMPO) Integrator, significantly reducing the number of force evaluations per simulated time unit by predicting forces at progressively larger intervals, thus boosting force-call efficiency. We incorporated the TEMPO integrator in a multiscale Brownian dynamics (MSBD) simulation tool. Compared with standard Brownian dynamics using the Euler-Maruyama integrator, our benchmarks of MSBD demonstrated 27- to 32-fold efficiency improvements for intrinsically disordered protein models and a seven-fold gain for nucleocytoplasmic transport through the nuclear pore complex (NPC), a critical cellular process in health and disease. Unlike conventional enhanced sampling, MSBD preserves kinetic properties, such as reaction rates, without relying on prior system knowledge or predefined reaction coordinates. By leveraging the inherently multiscale structure of energy landscapes, MSBD facilitates rapid molecular simulations while maintaining their accuracy. TEMPO’s flexible framework is generalizable to various biomolecular systems and could complement existing enhanced sampling methods, facilitating efficient exploration of energy landscapes or complex dynamical processes.

**Availability and implementation:**

https://github.com/ravehlab/tempo.

## 1 Introduction

Molecular dynamics (MD) simulations have become indispensable for characterizing various biomolecular processes linked to both health and disease (Lazim *et al.* 2020, Dal Lin *et al.* 2022). Originally conceived as a purely theoretical tool, MD simulations now provide practical insights into complex biomolecular processes and complement experimental methods, including in fields such as drug design (Poyraz *et al.* 2024), protein folding (Duan *et al.* 2019), macromolecular interactions (Dror *et al.* 2012), membrane dynamics (Goossens and De Winter 2018), ion channel gating (Bernèche and Roux 2005), enzyme catalysis (Warshel *et al.* 2006), and nanotechnology (Pasquinelli and Yingling 2016). As experimental techniques improve and simulation methodologies advance, there is a growing synergy between computation and experiment, leading to deeper insights and more precise models of molecular behavior (Orioli *et al.* 2020).

In a standard MD simulation, the system of interest is described by a set of particles in Cartesian space, exerting forces on one another and moving accordingly. The temporal trajectories of these particles are computed by numerically integrating Newton’s equations of motion, using small time steps to maintain numerical stability by capturing the fastest vibrational modes of motion, e.g. hydrogen vibrations. The resultant trajectories represent the thermodynamic ensemble of states that the system can occupy and also indicate the rates of transitions among these states (Hollingsworth and Dror 2018). However, unbiased MD simulations often suffer from infrequent transitions between system states, necessitating long simulation times to capture rare but important events. Thus, unbiased MD may become too slow to sample relevant states adequately, especially when the relevant biological phenomena unfold over larger time or length scales. The rate-limiting factor is often the small integration time step, driven by the need to resolve high-frequency motions such as bond vibrations while maintaining accuracy. As biological timescales of interest typically exceed the fast vibrational modes by many orders of magnitude, the large number of simulation steps needed drives up computational costs. Each step in the simulation requires at least one force calculation, which is computationally expensive (Yang *et al.* 2019).

Over the years, numerous methods have been proposed to alleviate these difficulties and accelerate MD simulations (Hénin *et al.* 2022). Existing methods include enhanced sampling strategies designed to bias sampling toward relevant states, thereby reducing the number of steps required to observe important events; coarse-grained techniques in which the dimensionality of the system’s configuration is reduced (Clementi *et al.* 2000, Liwo *et al.* 2005, Marrink *et al.* 2007, Bereau and Deserno 2009, Davtyan *et al.* 2012, Tribello *et al.* 2014); and neural network-based strategies that either learn coarse-grained representations or attempt to predict the evolution of the system without performing full dynamical simulations (Mardt *et al.* 2018, Noé *et al.* 2019, Arts *et al.* 2023).

Simulations can also be accelerated by reducing the number of the system’s degrees of freedom, either by substituting some of them with implicit stochastic terms or through spatial coarse graining. For example, in Langevin dynamics and Brownian dynamics (BD), solvent molecules accounting for most of the system’s degrees of freedom are substituted by an implicit stochastic term, which accounts for their random collisions with the solute (Schlick 2013). BD is particularly appealing in scenarios where inertia can be neglected and the system is dominated by random collisions and friction (Ermak and McCammon 1978). Furthermore, BD simulations often employ spatial coarse graining (Marrink *et al.* 2007), where each particle is represented at a coarse-grained level, further reducing the system’s dimensionality and thus allowing large systems to be studied more efficiently than fully atomistic MD. BD and spatially coarse-grained simulations have been successfully applied in multiple areas, including for complex processes such as nucleocytoplasmic transport through the nuclear pore complex (NPC) (Moussavi-Baygi *et al.* 2011, Timney *et al.* 2016, Kim *et al.* 2018, Winogradoff *et al.* 2022, Matsuda and Mofrad 2024, Raveh *et al.* 2024), which involve millions of atoms or more, and where they complement computationally expensive MD simulations of specific system parts at atomic resolution (Isgro and Schulten 2005, Raveh *et al.* 2016). However, even BD combined with spatial coarse graining can be limited by the cost of force function evaluations and the length of simulations needed to capture important phenomena such as nucleocytoplasmic transport.

Spatial coarse graining can be complemented with a parallel concept of temporal coarse graining. In essence, temporal coarse graining would seek to integrate over faster events so that longer time steps may be used to inform slower, more relevant motions. Such coarse graining is justified by the prevalence of local separation of scales in biomolecular systems, where fast, high-frequency processes often take place over short time and length scales, whereas slower dynamics such as conformational transitions, folding, or domain reorientations occur over longer periods (Karplus and McCammon 2002, Henzler-Wildman and Kern 2007). The fast fluctuations often manifest as noise that can be averaged out, letting the simulator devote fewer resources to them while still accurately capturing the slower dynamics. A classic and early example of a method that effectively carries out temporal coarse graining (and also impacts spatial degrees of freedom) is the SHAKE algorithm (Ryckaert *et al.* 1977, Macuglia 2023). By constraining the bond lengths of hydrogen atoms, SHAKE removes the necessity to explicitly resolve high-frequency vibrations, enabling a longer integration step. In effect, it “averages out” these small-scale, fast motions to focus on slower phenomena without overly compromising the overall physical fidelity of the simulation. Similarly, methods that compute a potential of mean force (PMF) (Torrie and Valleau 1977, Kumar *et al.* 1992, Darve and Pohorille 2001) leverage temporal coarse graining and local separation of scales by integrating out fast fluctuations of the energy landscape, but they often require prior information on the system. However, such methods typically lose the critical information on the system’s kinetics, require prior knowledge on, e.g. the reaction coordinates, and operate at specific spatiotemporal scales.

Here, we developed the Temporally Multiscale Prediction (TEMPO) integrator, a new integration algorithm that recursively implements temporal coarse graining. We hypothesize that temporal coarse graining, relying on the ubiquitous separation of scales in biological systems, allows a multiscale divide-and-conquer algorithmic strategy for simulations, which may operate more effectively across multiple spatiotemporal scales without assuming specific prior knowledge, all the while retaining kinetic information. In the TEMPO integrator, local samples from the force function are used to predict future force evaluations at a local neighborhood, informing future integration time steps without requiring explicit force evaluations. This process is repeated recursively, expanding the integrated time scale exponentially with the recursion depth relative to the time step at the recursion base. This approach eases the computational burden of force evaluations, a key factor limiting simulation times (Frenkel and Smit 2023), by reducing the number of force evaluations per time simulated, or force-call efficiency.

We evaluate the new TEMPO integrator using BD simulations that employ it internally, which we refer to as Multiscale BD (MSBD). We benchmark these simulations first on relatively simple coarse-grained models of intrinsically disordered peptides (IDPs), then on a model of nucleocytoplasmic transport through the NPC, a large channel where IDPs form a selective filter for nearly all biomolecular traffic between the cytoplasm and the nucleus. We compare the force-call efficiency of MSBD using TEMPO with naive BD simulations using a standard integrator and assess the retention of key thermodynamic and kinetic properties.

## 2 Methods

Algorithm 1 TEMPO($x_{0},\Delta t,n,f,s=4,T=298.15$)

**if**  n==0  **then**

  $x_{1}$, $F_{0\to 1}$, $R_{0\to 1}\leftarrow$  DoStepNaive($x_{0}$, Δt, *f*, *T*)

  **return**  $x_{1}$, $F_{0\to 1}$, $R_{0\to 1}$

**else**

  $x_{1},{\hat{F}}_{0\to 1},R_{0\to 1}\leftarrow$ TEMPO($x_{0}$, Δt/s, n−1, *f*, *s*, *T*)

  $x_{2},{\hat{F}}_{1\to 2},R_{1\to 2}\leftarrow$ TEMPO($x_{1}$, Δt/s, n−1, *f*, *s*, *T*)

  $R_{0\to 2}\leftarrow [R_{0\to 1},R_{1\to 2}]$

  $x_{s},{\hat{F}}_{0\to s},R_{0\to s}\leftarrow$

    DoStepPredicted( $x_{0},x_{1},\Delta t/(s^{n}),{\hat{F}}_{0\to 1},{\hat{F}}_{1\to 2},R_{0\to 2},s,n,T$ )

  **return**  $x_{s}$, ${\hat{F}}_{0\to s}$, $R_{0\to s}$

**end if**

### 2.1 The TEMPO integrator

**Figure 1.**
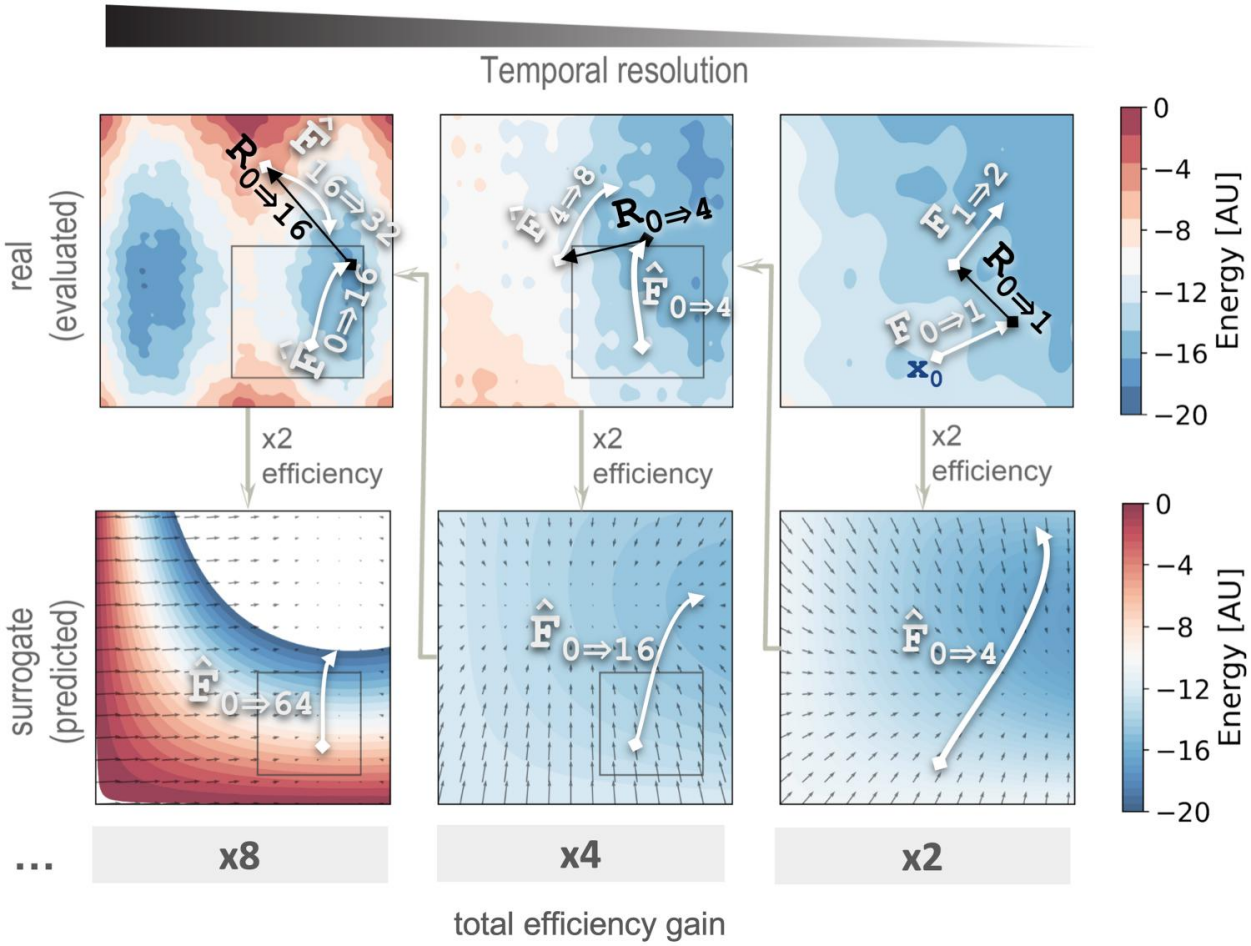
The Temporally Multiscale Prediction (TEMPO) integrator (Algorithms 1–3). Each recursion level (left to right) employs two shorter-timescale recursive calls to TEMPO, integrating from time step *i* to *j* (top row, $ֿ{\hat{F}}_{i\to j}$). At the base level (top-right), the algorithm uses the standard integrator over a single integration step (top-right, ${\hat{F}}_{i\to i+1}$.). These sampled force vectors are used to integrate the force over *s* time steps (here, *s *= 4), by computing a predicted surrogate energy function (bottom row), where the force is the gradient of the energy. Dark arrows indicate the implicit random component in Brownian or Langevin dynamics.

The principal assumption of our approach is that the dynamics of biomolecular systems span multiple time scales, arising from rugged energy landscapes containing nested minima (Karplus and McCammon 2002). Broadly speaking, the TEMPO integrator relies on a recursive algorithmic design, which applies progressively finer time resolutions. At each time resolution, TEMPO predicts forces over several future time steps based on recursively estimating the force over short time steps, corresponding to rapidly sampled modes (Algorithm 1; Fig. 1, left to right). It then passes on aggregated or averaged information to higher recursion levels, using the results from shorter time steps to predict the force over longer time steps (Fig. 1; right to left). This procedure is repeated up the hierarchy, allowing the method to integrate over large time steps using relatively few direct force evaluations. At the recursion base, we simply call the naive force function. By replacing force function calls at progressively higher recursion levels with locally predicted forces, TEMPO eases the computational burden of force evaluation exponentially with increasing recursion depth. For example, if two force estimates are used to predict the next two time steps (Fig. 1), then the proportion of direct force evaluation is halved for each unit of increasing recursion depth.

### 2.2 BD simulations using TEMPO

In the current implementation, the TEMPO algorithm (Algorithm 1) is adapted to integrate the BD stochastic differential equation of the form $\frac{dx}{dt}=-\frac{D}{k_{B}T}F(x)+\sqrt{2D}R(t)$ where x is the current system’s configuration, t is time, F(x) is the force vector, or the gradient of the underlying energy potential, D is the diffusion coefficient, and R(T) is white noise, implicitly representing random collisions with water molecules (Ermak and McCammon 1978, Schlick 2013). Over the discrete time step, the white noise is equivalent to sampling from the normal distribution with zero mean and standard deviation of $\sqrt{2D\Delta t}$. We refer to a BD simulation that employs the TEMPO integrator as multiscale Brownian dynamics (MSBD). In contrast, we refer to a BD simulation that employs a standard integrator as naive BD. We note that in principle, the same approach could be used for other kinds of simulations, e.g. by adapting the equations to account for momenta.

To integrate the BD stochastic differential equation more efficiently, with less calls to the force function, TEMPO performs a multiscale integration step of the force function f starting from an initial configuration x0 over a total time step of *Δt*, refined across scales by the factor s at each recursion level, up to recursion depth n. To advance the simulation at each recursion level, TEMPO calls DoStepPredicted (Algorithm 2), which relies on force predictions from lower recursion levels, corresponding to shorter time steps, to predict the force over longer ones. To predict forces in a neighborhood of the current configuration, it invokes GetSurrogateForce (Algorithm 3). The surrogate force calculation merges a mean force term ($F_{\mathrm{Mean}})$, which averages out high-frequency noise, and a local Taylor expansion term ($F_{\mathrm{Taylor}})$, which predicts the short-range trend in the force, using a normalization step to limit the magnitude of this term and prevent overshooting. A hyperparameter α controls the weight of each term. Together, these terms balance smoothed forces with short-range trend prediction.

Algorithm 2 DoStepPredicted$\left(x_{0},x_{1},\Delta t,{\hat{F}}_{0\to 1},{\hat{F}}_{1\to 2},R_{0\to 2},s,n,T\right)$

**Step 1: Expand *R* to match size**  $s^{n}$

$R_{0\to s}\leftarrow R_{0\to 2}$

**while**  $\text{len}(R_{0\to s})<s^{n}$  **do**

   r←GetSurrogateForce(0, $\sqrt{2\cdot D\cdot \frac{\Delta t}{s^{n}}}$)

  $R_{0\to s}.\text{append}(r)$

**end while**

**Step 2: Surrogate force calculation and updates**

$x_{\text{cur}}\leftarrow x_{0}$

${\hat{F}}_{0\to s}\leftarrow [0,\ldots ,0]$

**for** *i* in range($s^{n}$) **do**

  $SF_{i\to i+1}\leftarrow$  GetSurrogateForce($F_{0\to 1},F_{1\to 2},x_{0},x_{1},x_{\text{cur}}$)

  $x_{\text{cur}}\leftarrow x_{\text{cur}}+\frac{D\cdot \Delta t}{k_{B}T}SF_{i\to i+1}+R_{0\to s}[i]$

  ${\hat{F}}_{0\to s}\leftarrow {\hat{F}}_{0\to s}+SF_{i\to i+1}/s^{n}$

**end for**

**Step 3: Update position at final recursion level**

$x_{s}\leftarrow x_{\text{cur}}$

**Return:**  $x_{s}$, ${\hat{F}}_{0\to s}$, $R_{0\to s}$

Algorithm 3 GetSurrogateForce $(F_{0\to 1},F_{1\to 2},x_{0},x_{1},x_{\text{cur}},\Theta_{\text{normalize}}=\text{SIMPLE},\alpha =0.5)$

$F_{\text{Mean}}\leftarrow \frac{F_{0\to 1}+F_{1\to 2}}{2}\;$

$F_{\text{Taylor}}\;\leftarrow \;F\left(x_{0}\right)+\left(x_{\text{cur}}-x_{0}\right){}^{\circ}\frac{F_{1\to 2}-F_{0\to 1}}{\left(x_{1}-x_{0}\right)}$

**if**  $\Theta_{\text{normalize}}=\text{SIMPLE}$  **then**

 $F_{\text{Taylor}}\;\leftarrow \;\frac{F_{\text{Taylor}}}{\left\|F_{\text{Taylor}}\right\|}$

**else**

 $F_{\text{Taylor}}\;\leftarrow \;\frac{\min\left(\left\|F_{\;\text{Mean}}\|,\|F_{\text{Taylor}}\|,\|{\hat{F}}_{0\to 1}\|,\|{\hat{F}}_{1\to 2}\right\|\right)}{\left\|F_{\text{Taylor}}\right\|}\cdot F_{\text{Taylor}}$

**end if**

$F_{\text{Surrogate}}\;\leftarrow \;\alpha F_{\text{Mean}}+\left(1-\alpha \right)F_{\text{Taylor}}$

**return**  $F_{\text{Surrogate}}$

### 2.3 IDP polymer models

**Figure 2.**
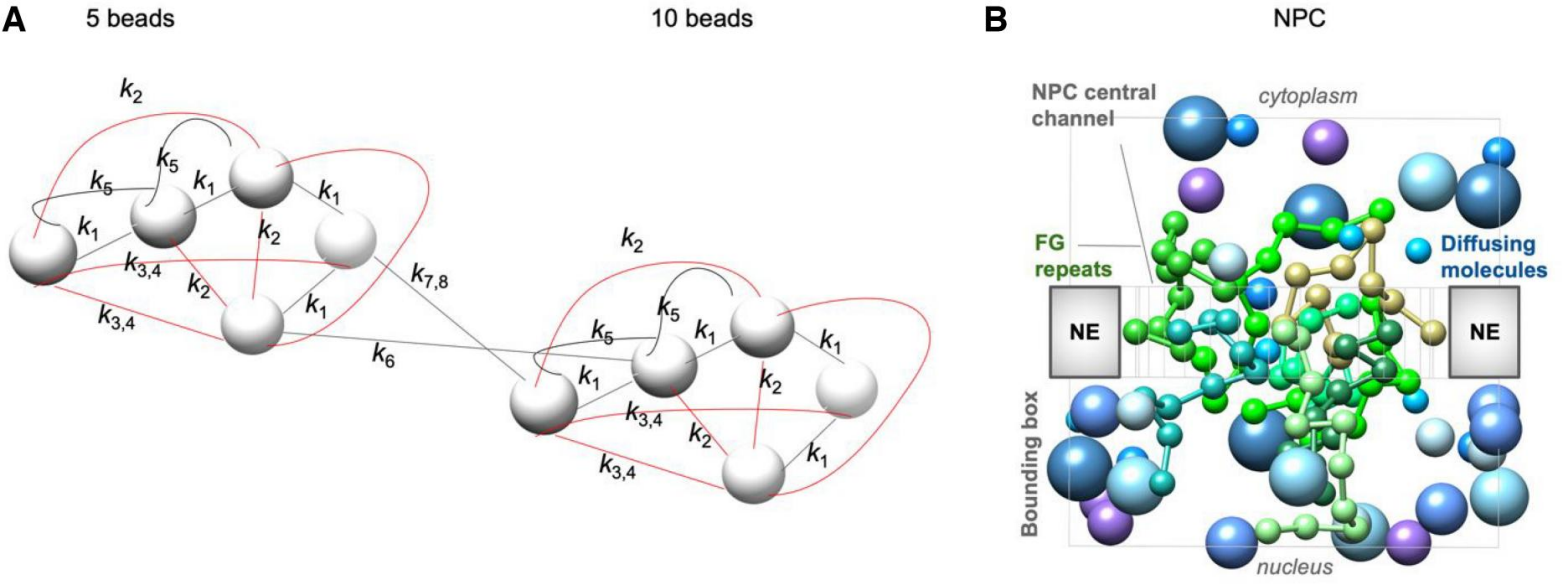
A visual representation of the benchmark systems. (A) 5-bead and 10-bead flexible polymer models of intrinsically disordered proteins (IDPs), using the classic beads-on-a-string model of polymers. Beads are bonded by harmonic distance restraints (black) and non-bonded interactions are modeled with truncated-harmonic distance restraints (red). The 5-bead system is a subset of the 10-bead system. Note a bead does not represent an atom but rather a link in a flexible polymer chain (e.g. several consecutive residues). Each bead is treated equally, and the force coefficient of the spring is represented by *k_1_* through *k_n_*, respectively. Due to space considerations, the precise parameters and restraint types for these interactions are detailed in the code repository accompanying this manuscript. (B) The NPC model is represented similarly to our previous Brownian dynamics simulations of passive diffusion through the NPC (Timney *et al.* 2016, Kozai *et al.* 2023, Raveh *et al.* 2024) but uses a downsized representation for benchmarking purposes. The green strings-of-beads represent the various FG repeat proteins, which are anchored to the NPC scaffold walls, and the gray beads represent the passively-diffusing molecules. The NPC scaffold is represented as a cylindrical cavity in the nuclear envelope.

IDPs were represented as coarse-grained bead-on-a-string (Fig. 2A; Supplementary Methods). Each bead was connected to its neighbors by harmonic distance restraints, set to match the expected bond lengths. We optionally added bending restraints to control local stiffness. Longer-range or non-bonded interactions were modeled by weaker harmonic or truncated-harmonic potentials. The spring constants were scaled by thermal energy (298.15 K) so that fluctuations remained physically realistic. The diffusion coefficient was set to 1 ${Å}^{2}s^{-1}$. We note that the physical values in these models are not intended as realistic representations but rather as toy model parameters for benchmarking MSBD and TEMPO, and that the reported timescales are not intended as absolute physical values, but rather relative values normalized by the input diffusion coefficients (Supplementary Tables 1–3).

### 2.4 NPC representation and components

Our model of the NPC (Fig. 2B) is a downsized variant of our previous Brownian dynamics simulations of passive diffusion (Timney *et al.* 2016, Kim *et al.* 2018, Kozai *et al.* 2023, Raveh *et al.* 2024). A flat slab of height 55 or 110 Å represents the nuclear envelope (NE), with a cylindrical pore of radius 90 Å at *z *= 0. In this downsized representation, either one or three layers, each comprising eight chains of intrinsically disordered FG repeats. Each chain is anchored to the cylinder walls, and it is represented using eight beads and an anchor bead attached around the pore’s inner surface. The radius of each bead was set to 8 Å, representing a “fuzzy” distribution over the atomic coordinates of 20 intrinsically disordered FG repeat residues (Raveh *et al.* 2016, Timney *et al.* 2016, Raveh *et al.* 2024). 35 passively diffusing molecules of radius 8–20 Å, corresponding to molecular weights of 2–27 kDa, were placed at random positions at the upper half of the NPC, representing the cytoplasm. All particles were confined within a 130 × 130 × 130 Å³ or 260 × 260 × 260 bounding box. Before follow-up statistical analysis, the model was equilibrated for 1 µs using a step size of 2000 fs at room temperature using a naive BD integrator.

### 2.5 Interactions and restraints

The global excluded-volume term prevents bead overlap with *k *= 1 kcal/mol/Å^2^. Adjacent FG beads were connected by harmonic distance restraints (rest length = 30.4 Å, *k *= 2.0 kcal/mol/Å^2^). Non-bonded interactions were represented using truncated-harmonic terms (rest length = 3.0 Å, *k *= 0.002 kcal/mol/Å^2^, threshold = 5.0 Å), which captured weaker non-bonded interactions. The bounding box was enforced using IMP’s bounding box harmonic restraint with *k *= 2 kcal/mol/Å^2^. The NE barrier was enforced using a harmonic restraint with *k *= 5 kcal/mol/Å^2^.

## 3 Results

We now describe the application of the TEMPO integrator, specifically in the context of BD simulations, where inertial terms are ignored in favor of diffusion and friction. We benchmark MSBD, which incorporates the TEMPO integrator, first on flexible polymer models representing IDPs and then on a simplified model of nucleocytoplasmic transport through the NPC.

### 3.1 Five-bead IDP model

We first examine a variant of the classic beads-on-a-string polymer model, where each consecutive pair of beads is connected by a spring, and non-bonded interactions are sometimes introduced to mimic intramolecular cohesive effects (Flory 1953, Van Der Maarel 2007, Kozai *et al.* 2023, Raveh *et al.* 2024) (Fig. 2A). This simple model can reproduce a wide range of behaviors seen in IDPs. We used a gold-standard BD simulation with a small time step, 0.02 fs, to collect reference data about the distribution of the polymer’s radius of gyration (*R_g_*) and its relaxation kinetics (e.g. half-life of *R_g_* autocorrelation). We note this time step is artificially smaller than standard MD or BD simulations, because we use a custom force field for testing purposes, focusing on relative speedup gains rather than absolute units or realistic predictions.

**Figure 3.**
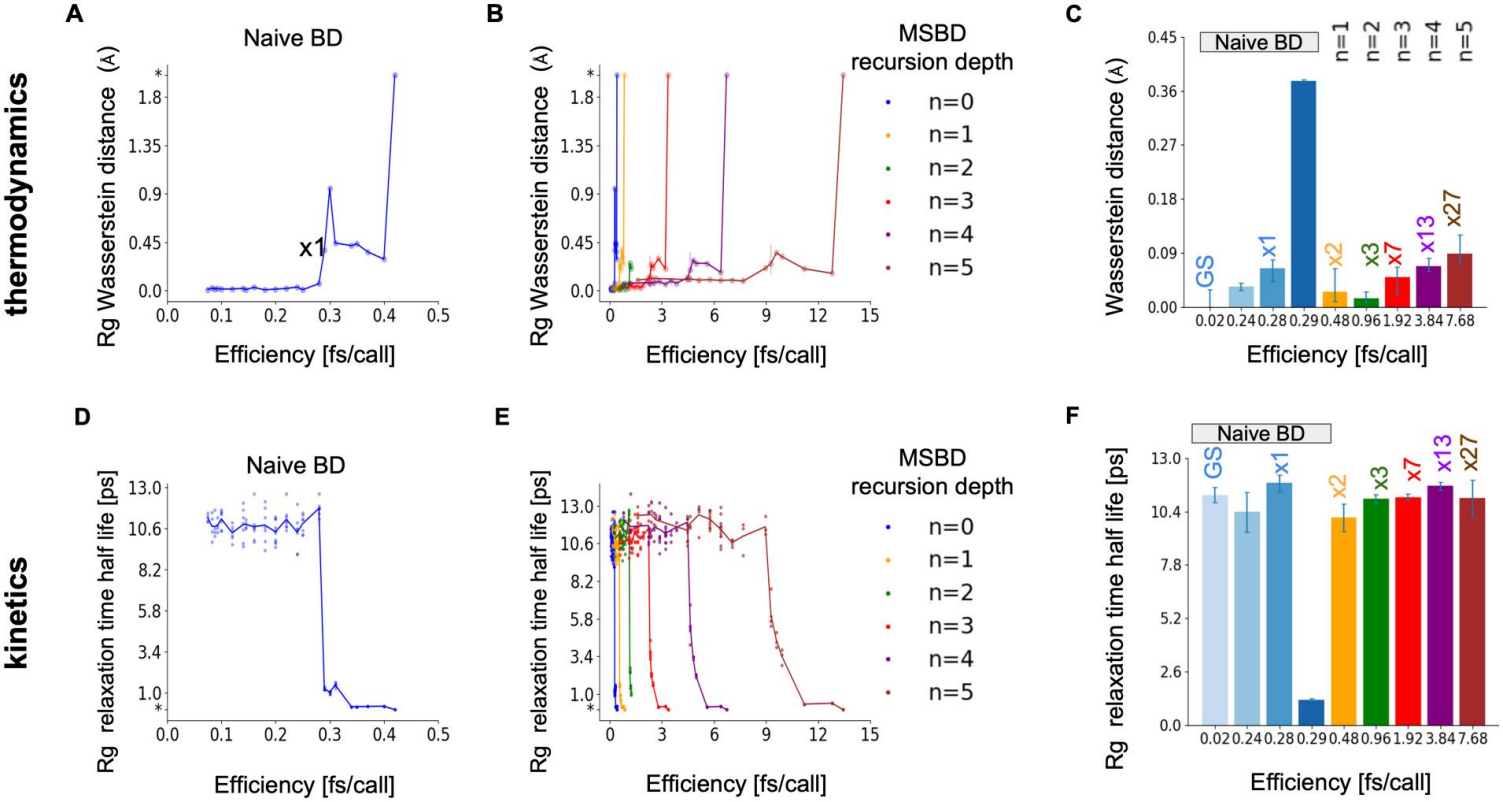
Thermodynamic and kinetic accuracy of MSBD using TEMPO and naive BD using a standard integrator at different force-call efficiency levels for a five-beads-on-a-string model of an IDP. (A) Thermodynamic error, measured as the mean Wasserstein distance of *R_g_* from the baseline gold-standard simulation, plotted against force-call efficiency for naive BD. Each data point is the mean distance over at least three independent runs, resulting in negligible error bars. The asterisk at the top of the *y*-axis indicates a loss of numerical stability. (B) Same as in A, for MSBD using different recursion depths (*n*) with *n* = 0 corresponding to naive BD. (C) Maximal speedup with tolerable loss of thermodynamic accuracy for different simulation setups with different force-call efficiencies (*x*-axis). Blue shades indicate naive BD using different force-call efficiencies. Orange, green, red, purple, and black bars indicate MSBD using recursion depths from *n *= 1 to *n *= 5; GS = gold standard simulation; naive BD with force-call efficiency of 0.29 fs/call is numerically stable but reaching a non-tolerable error level. (D) *R_g_* relaxation half-life, a property of the system kinetics, plotted against force-call efficiency. Each data point represents an independent run, and the line indicates their mean values. The asterisk at the bottom of the *y*-axis indicates loss of numerical stability. (E) Same as in D but for MSBD, using different recursion depths as in B. (F) Speedup versus means results as in C, but for the *R_g_* relaxation time.

To quantify how far a simulation deviates from this gold-standard ensemble of step size of 0.02 fs, we measure the Wasserstein distance (Earth mover’s distance) between the distributions of *R_g_* (Fig. 3A–C). We also examine the half-life extracted from autocorrelation curves, comparing it with the gold standard (Fig. 3D–F). We find that naive BD remains accurate up to a force-call efficiency level of about 0.28 fs/call, but beyond that level, both thermodynamics and kinetics begin to deviate from their reference gold standard values. In contrast, MSBD remains accurate at about 7.68 fs/call, representing a 27-fold improvement over naive BD.

### 3.2 Ten-bead disordered protein model

**Figure 4.**
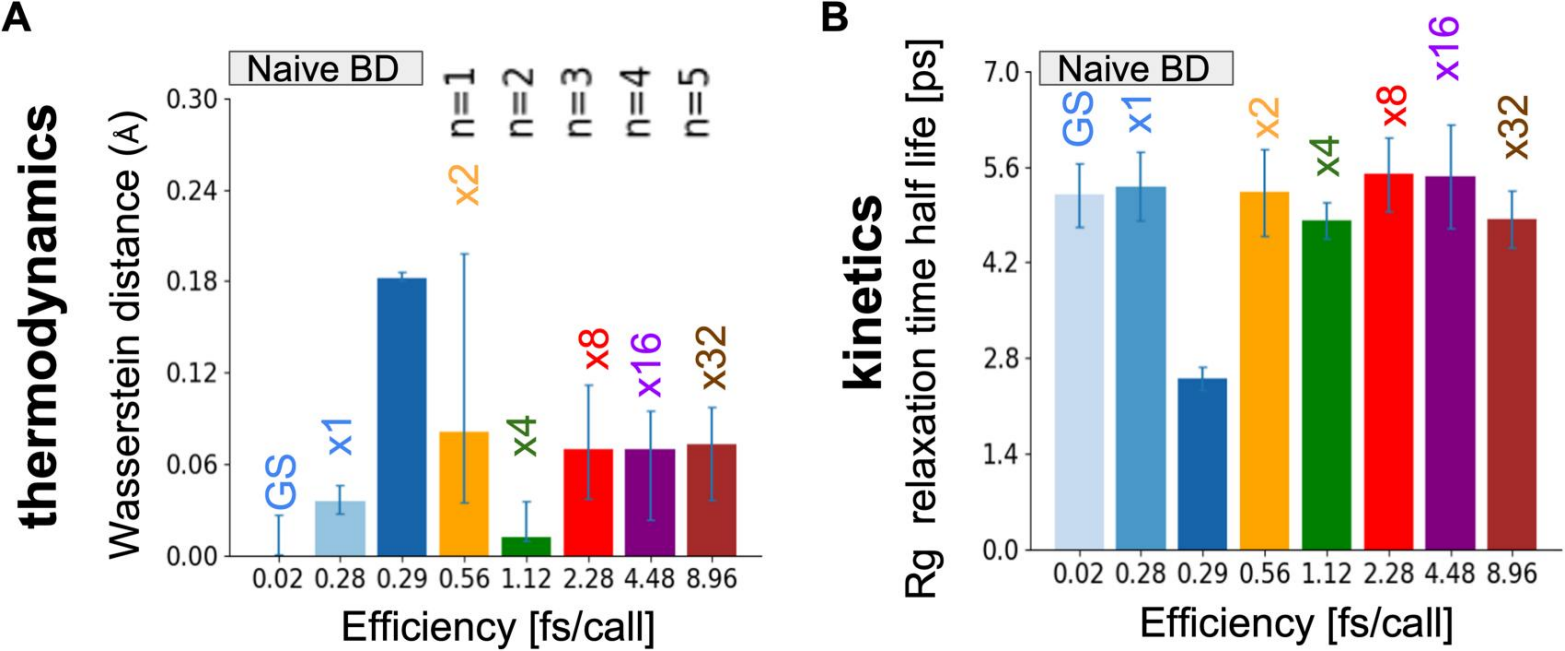
Thermodynamic and kinetic accuracy for the 10-bead disordered protein system. (A, B) Same legend as in Fig. 3C and F. In both 5- and 10-bead models, we did not test MSBD with a recursion depth of *n* = 6, because the *R_g_* relaxation time is faster than a single step.

To test how MSBD scales with system size, we use a similar polymer model containing 10 beads instead of five (Fig. 2A). For the baseline gold-standard BD, we use naive BD with a time step of 0.02 fs, observing that the time step can be pushed to around 0.28 fs/call before the simulation accuracy begins to degrade. MSBD holds accurate up to about 9 fs/call, producing an efficiency gain of roughly 32 times over naive BD (Fig. 4). This outcome reinforces the idea that local partial sampling and Taylor-based predictions remain valid for somewhat larger polymeric systems, as long as the local scale separation is retained.

### 3.3 Nuclear pore complex (NPC) model

Finally, we demonstrate that TEMPO can be used for simulating a realistic and highly complex biological system (Timney *et al.* 2016, Kim *et al.* 2018, Raveh *et al.* 2024), also serving as a model system for simulating complex systems containing IDPs (Banani *et al.* 2017, Cao *et al.* 2024). At 50–100 MDa, the nuclear pore complex (NPC) is one of the largest molecular assemblies in eukaryotic cells, comprising hundreds of protein subunits (Weis 2002, Hoogenboom *et al.* 2021, Cowburn and Rout 2023). Its central channel is formed by intrinsically disordered FG repeats that confer selective transport properties, allowing some molecules to pass while restricting others, with lively scientific debates regarding the molecular mechanism underlying its function (Hoogenboom *et al.* 2021). Our previous coarse-grained BD models of the NPC have demonstrated good predictive power for both passive and facilitated diffusion (Timney *et al.* 2016, Kim *et al.* 2018, Raveh *et al.* 2024). However, such simulations are computationally expensive because they require many force function evaluations and must be run over relatively long times to capture the relevant transport events.

We simulated transport through the NPC (Fig. 2B) with MSBD versus naive BD using a range of time steps and MSBD recursion depths, comparing the distribution of FG repeat *R_g_* values, the half-life of their autocorrelations, the passive diffusion rates of various-sized cargoes, and the energy barriers along the pore axis.

**Figure 5.**
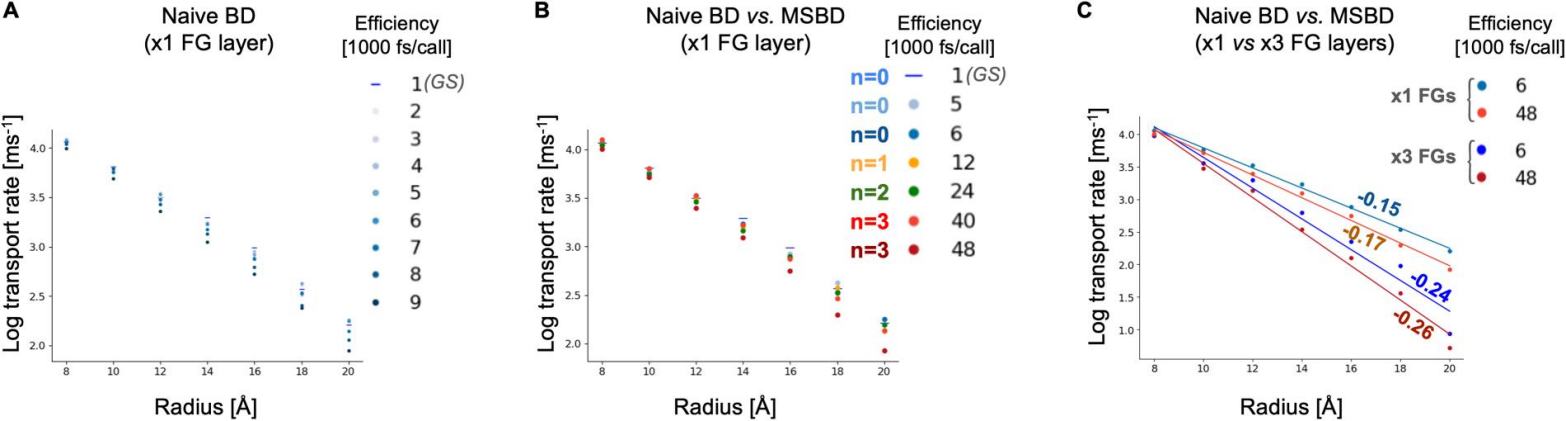
Kinetic analysis showing the mean passive transport rates over 50–80 independent simulations (*y*-axis; natural logarithm) as a function of the molecular radius of the passively diffusing molecules (*x*-axis), shown for: (A) naive Brownian Dynamics (BD) with different force-call efficiencies (brightness) for an NPC with one layer of FGs; GS = Gold Standard simulation. (B) Naive BD (blue hues; *n *= 0) versus MSBD using different recursion depths (hues) and force-call efficiencies (brightness) for an NPC with one layer of FGs; and (C) Naive BD (blue hues; *n *= 0) versus MSBD with recursion depth *n *= 3 (orange hues), which is eight times more force-call efficient, for an NPC with either one layer (bright) or three-layers (dark) of FG repeats, with a larger bounding box and nuclear envelope thickness (Supplementary Table 4). The lines represent exponential fits relating transport rates and radius (Raveh *et al.* 2024), along with the exponential decay constant in units of Å-1, as demonstrated previously (Timney *et al.* 2016; Raveh *et al.* 2024). NPC = nuclear pore complex.

**Figure 6.**
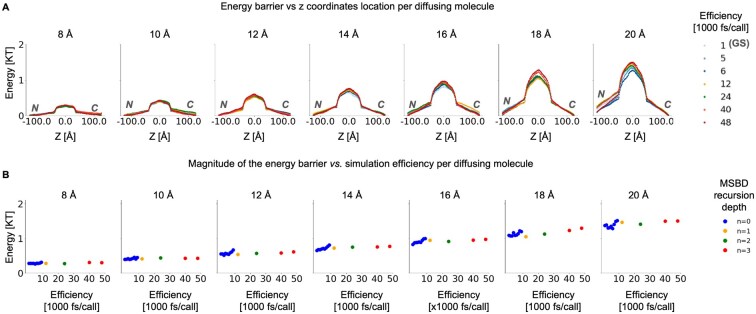
Thermodynamics analysis of the simulated free energy barrier along the NPC’s central axis. (A) Free energy for passively diffusing molecules under MSBD and naive BD, as a function of position along the central axis of the NPC’s central channel, running from the cytoplasm (“C”) to the nucleus (“N”), for different radii of the diffusion molecules (left to right panels). Each series is computed by averaging the density distribution along the *z*-axis in 60–80 independent simulations and applying a Boltzmann inversion, as in Timney *et al.* (2016) and Raveh *et al.* (2024). (B) The magnitude of the free energy barrier for transport is plotted against force-call efficiency for the same molecules as in A.

We found that the accuracy of naive BD deteriorates once the attempted force-call efficiency surpasses 6000 fs/call, losing all numerical stability beyond 9000 fs/call. In contrast, MSBD can be pushed to about 40 000 fs/call or more before it exhibits substantial inaccuracies in both kinetic and thermodynamic observables, yielding about a seven-fold improvement in force-call efficiency. Importantly, MSBD preserves an exponential relationship between passive diffusion rate and cargo radius, consistent with earlier theoretical and experimental data on passive diffusion through NPCs (Timney *et al.* 2016, Raveh *et al.* 2024) (Fig. 5) as well as the profiles of the free-energy barriers for passive diffusion (Fig. 6). To further evaluate TEMPO’s applicability to biologically relevant systems, we simulated an expanded NPC model containing three layers of FG-repeat regions, along with an increased bounding box and larger pore height (Fig. 5c). As with the smaller-scale system, the naive BD algorithm produced stable statistics at a force-call efficiency of 6000 fs/call, but lost numerical stability beyond 9000 fs/call, whereas TEMPO produces accurate results at 48 000 fs/call, yielding an eight-fold improvement in force-call efficiency. These differences reflect the well-established empirical and theoretical effects observed in deletion mutants in the real NPC or in artificial mimics with lower FG-repeat densities, which may result in higher leakage (Timney *et al.* 2016, Raveh *et al.* 2024). In other words, MSBD robustly reproduces the key kinetic and thermodynamic properties of the nucleocytoplasmic transport system. These tests demonstrate MSBD’s effectiveness and generality in a critical and ubiquitous biological system that exhibits complicated, multi-scale dynamics and a high dimensionality of interactions.

## 4 Discussion

We have introduced TEMPO, an integration algorithm based on temporal-multiscale force prediction. For simple polymer models of IDPs, BD simulations using this integrator, or MSBD, achieve speedups of 27- to 32-fold relative to naive BD simulations while maintaining the accuracy of both thermodynamic (e.g. the distribution of *R_g_*) and kinetic (e.g. relaxation rates) properties. For the more complex NPC model, MSBD reaches about a seven-fold improvement in force-call efficiency yet yields cargo diffusion rates and free-energy profiles comparable to gold-standard naive BD simulations, showcasing its utility for real-world characterization of biomolecular systems.

A key concept in our multiscale integration framework is local scale separation. Biomolecular systems often feature high-frequency fluctuations that can be regarded as noise from the vantage point of slower, large-scale transitions (Gunawardena 2014). By utilizing a recursive structure, MSBD systematically offloads the burden of resolving these fast fluctuations to lower recursion levels. The higher recursion levels then rely on surrogate predictions (Fig. 1), built from local expansions and partial sampling, to compute the forces for the next few steps without direct calls to the force function. The NPC example highlights the importance of this principle. Although the NPC includes intrinsically disordered FG-repeat regions that undergo rapid, localized changes, these high-frequency motions do not substantially perturb the macroscopic rates of transport through the pore, which evolve on longer timescales. Hence, it is sufficient to sample the smaller-scale dynamics at a finer resolution and then pass aggregated force data to the coarser (longer timescale) steps.

Another key component in our work is a surrogate function for predicting future forces based on local force prediction. In future work, the predicted surrogate force function will be refined and optimized, for instance, to include second-order derivatives. Furthermore, machine learning schemes can be used to learn the surrogate function either by training them in advance using deep neural networks (Alzubaidi *et al.* 2021, Wu *et al.* 2023) or adapting their parameters on-the-fly using, for example, reinforcement learning (Shakya *et al.* 2023). For example, a graph neural network could be employed to capture particle interactions more accurately, and an error-correction mechanism, informed by on-the-fly sampling of a naive force function, could detect and rectify emerging discrepancies in real time. These enhancements not only have the potential to boost simulation speed but also enable more effective parallelization of force evaluations.

In the current work, we focused on demonstrating the utility of TEMPO in accelerating coarse-grained models involving multiple IDP chains in crowded milieu and their interactions with globular proteins, using the NPC as a model system. Despite recent advances (Moussavi-Baygi *et al.* 2011, Timney *et al.* 2016, Winogradoff *et al.* 2022, Dekker *et al.* 2024, Raveh *et al.* 2024, Kreysing *et al.* 2025), simulating the NPC over sufficient timescales is a challenge long deemed intractable due to its large scale (millions of atoms, even without accounting for the solvent) and intricate dynamics (multiple components interacting across multiple spatiotemporal scales). The FG-repeat domains found in nucleoporins offer a broadly relevant model for simulating IDPs and biomolecular condensates well beyond the NPC. These repetitive motifs appear in various cytoplasmic and nuclear proteins, enabling the polymer-physics insights gleaned from FG-Nup studies to inform general principles governing IDP-mediated phase separation (Weis 2002, Banani *et al.* 2017, Hoogenboom *et al.* 2021, Shinkai *et al.* 2021, Cowburn and Rout 2023, Cao *et al.* 2024), from selective barriers to biomolecular condensate assembly. Although our current exploration highlights coarse-grained models, TEMPO is not restricted to these representations.

We next discuss several limitations of the current version of the TEMPO integrator and how to address them. First, in its current iteration, TEMPO was tailored for Brownian dynamics. Nonetheless, as its algorithmic core relies on recursive force predictions from local samples, it can thus be adapted for any type of molecular dynamics simulation, and in principle, for simulating any type of dynamical system evolving in a continuous energy landscape. Specifically, TEMPO may be adapted to fully atomistic simulations. However, the more rugged energy landscapes and correlated motions characteristic of all-atom systems might require further tuning, forming a promising avenue for our future work. In the future, we will also explore whether TEMPO can be extended to systems with complex coupling between local and global dynamics, for instance, by adaptively adjusting recursion depth or partial sampling protocols on-the-fly. Second, hyperparameters such as the recursion depth and the simulation time step must be chosen carefully, considering both system-specific optimization, such as the time step for the simulation, and the maximum permissible error. We address this limitation by proposing a practical and empirically-tested heuristic for choosing the time step and recursion depth, balancing accuracy and speed, and addressing system-specific adaptations (Supplementary Material, Heuristic for Choosing Hyperparameters). Third, overly deep recursions may accumulate significant inaccuracies if the local expansions or partial sampling do not capture essential cross-terms in the force, potentially leading to overshooting or undershooting. As discussed above, such inaccuracies can be addressed by adding a layer of quality control to identify and correct accumulated numerical errors. Lastly, from a more practical point of view, our current Python implementation might limit large-scale performance. We are currently developing a C++ and GPU-based version, which would enable more comprehensive production runs, also benefiting from the inherently parallelizable nature of our temporally multiscale force prediction scheme.

## 5 Conclusion

We have presented the temporally multiscale prediction (TEMPO) integrator and MSBD that employ this integrator. TEMPO leverages temporal coarse graining via recursive integration to accelerate Brownian dynamics simulations. By predicting the force function in a local neighborhood from localized force samples, Taylor expansions, and force averaging, the TEMPO integrator cuts down on expensive force function calls while preserving thermodynamic and kinetic integrity. Our method complements rather than supplants existing accelerated sampling or coarse-graining approaches. In principle, the temporally multiscale force prediction approach of TEMPO can be straightforwardly adapted and integrated with these methods to further expand the range of accessible timescales, for instance, in the context of free energy calculations. Given the prevalence of local scale separation in many biomolecular and soft-matter systems, we anticipate that TEMPO will be a foundational tool, alongside existing enhanced sampling and coarse-graining techniques, for reaching the spatiotemporal regimes critical to understanding complex biological phenomena.

## Supplementary Material

vbaf142_Supplementary_Data

## Data Availability

The data underlying this article are available in https://github.com/ravehlab/tempo. Supplementary data are available at https://github.com/ravehlab/tempo.
