# Supplementary material for: The TEMPO integrator: accelerating molecular simulations by temporally multiscale force prediction
Source: Bioinform Adv. 2025 Jun 20;5(1):vbaf142. doi: 10.1093/bioadv/vbaf142 (PMC12377907; doi:10.1093/bioadv/vbaf142)
Supplement: Supplementary file 1 [file vbaf142_supplementary_data.zip › Supp_TEMPO_v2.odt]

Supplementary Appendix

 The TEMPO Integrator: Accelerating Molecular Simulations by Temporally-Multiscale Force Prediction

Reshef Mintz1, Barak Raveh1

1 School of Computer Science and Engineering, Hebrew University of Jerusalem, Jerusalem, Israel.

Keywords: Multiscaling, Temporal Coarse-Graining, Brownian dynamics, Nucleocytoplasmic Transport, Intrinsically Disordered Proteins, Molecular  dynamics simulations

Table of Contents

[Supplementary Methods.	2](#_1z6kwaiwsrje)

[5- and 10-bead models	2](#_1ktk6vrklmhy)

[Model components	2](#_qrd4mwrjifmw)

[Model interactions	3](#_qd22c5bve66p)

[The Nuclear Pore Complex (NPC) model	5](#_svfxe9k72oso)

[Model components	5](#_jw0zwrjrkk65)

[Equilibration.	6](#_hspuzajed9gt)

[Heuristic for choosing hyperparameters.	8](#_aomkhwmpq5de)

[Supplementary References.	10](#_qffu2paz5oz)

Supplementary Methods.

All models were implemented in the open-source Integrative Modeling Platform (IMP) software ([http://integrativemodeling.org](http://integrativemodeling.org)) [(Russel ](https://paperpile.com/c/Hr0OH4/Qocn+oWaGq+fiIwj)[et al.](https://paperpile.com/c/Hr0OH4/Qocn+oWaGq+fiIwj)[, 2012; Sali, 2021; Rout and Sali, 2019)](https://paperpile.com/c/Hr0OH4/Qocn+oWaGq+fiIwj). 

Code and Data availability: [https://github.com/ravehlab/tempo](https://github.com/ravehlab/tempo)

5- and 10-bead models

Model components

Figure S1. The 5-bead model. A visual representation of the 5-bead flexible polymer model of an intrinsically disordered protein, using a classic beads-on-a-string approach, augmented with non-bonded interactions. Each pair of consecutive beads (a–e) is bonded via a harmonic restraint (black), while nonbonded interactions are handled by a truncated harmonic potential (red). A single bead represents a link in the polymer chain (e.g., several consecutive residues) rather than a single atom. The force coefficients of the springs are labeled k1 through kn (Table 3). The notation ki,j​ is used to indicate that it encompasses both ki​ and kj​ e.g k3,4 mean two forces are applied one with a spring constant of  k3  and the other with a spring constant of k4

Figure S2. The 10-bead model. A visual representation of the 10-bead flexible polymer model of an intrinsically disordered protein, following a classic beads-on-a-string approach, augmented with non-bonded interactions. Consecutive beads are bonded via harmonic restraints (black), while nonbonded interactions are represented by truncated harmonic potentials (red). Each bead, labeled a–e and a′–e′, represents a coarse-grained link (e.g., several consecutive residues) rather than an individual atom. The force coefficients of the springs are labeled k1 through kn (Table 3). The notation ki,j​​ is used to indicate that it encompasses both ki​ and kj​ e.g k3,4 mean two forces are applied one with a spring constant of  k3  and the other with a spring constant of k4

Polymer chains were represented using the beads-on-a-string model. Each pair of beads was connected by a flexible spring. The beaded radii were uniformly set to 1 Å; we note that this relatively small size is an arbitrary choice that we used for benchmarking MSBD on the 5-bead and 10-bead toy systems (Table 1), and it is not intended as a realistic physical representation. The interactions between consecutive beads were modeled using harmonic potentials with varying spring constants to capture heterogeneous interaction strengths typical of real-world systems (Table 2-3).

Model interactions

The primary bonded interactions between consecutive beads in the chain were defined by the parameters of specific forces (Table 3, first row), ensuring the polymer's structural integrity. Additional local angular or bending interactions within the chain were modeled using the parameters in the fourth row, emphasizing flexibility. Long-range interactions between non-consecutive beads within the same chain were represented using parameters in the fifth and sixth rows, capturing reduced interaction strengths for distant bead pairs. Interactions reflecting steric constraints or specific long-range associations were modeled using parameters from the last two rows, where more substantial constraints were applied due to larger radii or specific structural properties.

The harmonic potentials for these interactions were implemented using IMP’s HarmonicDistancePairScore and SphereDistancePairScore, with parameters specific to each interaction type, as described in the table. The simulation was conducted at room temperature (T=298.15K), and the spring constants were scaled based on thermal energy. 

| Component | Relevant IMP classes | Main parameters | Comments |
| --- | --- | --- | --- |
| simple polymer models | Particle,  XYZR,  Diffusion | Bead radius: 1 Å Beads per chain (excluding anchor bead): 5-10 Diffusion coefficient: computed by Stokes’ law using the default by IMP | Each chain is represented as a flexible string of beads. |

Table 1. Summary of the simple polymer model components, including technical representation in the Integrative Modeling Platform (IMP)[(Russel ](https://paperpile.com/c/Hr0OH4/Qocn)[et al.](https://paperpile.com/c/Hr0OH4/Qocn)[, 2012)](https://paperpile.com/c/Hr0OH4/Qocn).  

| Forces | Relevant IMP classes | Main parameters | Potential function [kcal/mol] |
| --- | --- | --- | --- |
| specific | IMP.core. HarmonicDistance PairScore | x0 - rest length in Å k - force coefficient in kcal/mol/Å2 | 0.5k∙(d-x0)2 |
| non-specific | IMP.core.Sphere  DistancePairScore (IMP.core.Truncated HarmonicUpperBound) | x0 - rest length in Å k - force coefficient in kcal/mol/Å2 θ - upper threshold in Å | if x<x0:   0   if x0≤x≤(x0+θ):    0.5∙k(dsphere-x0)2 if x>(x0+θ):    converge to a limit |

Table 2.  Summary of forces used in the polymer model, including technical representation in the Integrative Modeling Platform (IMP) [(Russel ](https://paperpile.com/c/Hr0OH4/Qocn)[et al.](https://paperpile.com/c/Hr0OH4/Qocn)[, 2012)](https://paperpile.com/c/Hr0OH4/Qocn). d indicates the distance between bead centers, dsphere indicates the distance between their surfaces.

| Restraint name | IMP Restraint type | Parameters  (see Table 2) | Restrained pairs of beads |
| --- | --- | --- | --- |
| k1 | IMP.core. HarmonicDistancePairScore | x0 = 4 k = T_k*1.8 | a-b, b-c, c-d, d-e a-b, b’-c’, c’-d’, d’-e’ |
| k2 | IMP.core. SphereDistancePairScore (IMP.core.Truncated HarmonicUpperBound) | x0 = 10 k = T_k Θ = 11 | a-c, b-d, c-e a,-c,, b,-d,, c,-e, |
| k3 | IMP.core. SphereDistancePairScore (IMP.core.Truncated HarmonicUpperBound) | x0 = 8 k = T_k/16 Θ = 13 | a-e, a-d a’-e’, a’-d’ |
| k4 | IMP.core. HarmonicDistancePairScore | x0 = 12 k = T_k/16 | a-e, a-d a’-e’, a’-d’ |
| k5 | IMP.core. HarmonicDistancePairScore | x0 = 17 k = T_k/64 | a-b, b-c a’-b’, b’-c’ |
| k6 | IMP.core. HarmonicDistancePairScore | x0 = 4 k = T_k*18 | a-b, e-a’ |
| k7 | IMP.core. HarmonicDistancePairScore | x0 = 12 k = T_k/16 | e-b’, d-a’ |
| k8 | IMP.core. HarmonicDistancePairScore | x0 = 17 k = T_k/64 | d-a’ |

Table 3. Restraint types and parameters for the 5-bead and 10-bead systems (Figure 2, S1, S2), including technical representation of these restraints in the Integrative Modeling Platform (IMP) [(Russel ](https://paperpile.com/c/Hr0OH4/Qocn)[et al.](https://paperpile.com/c/Hr0OH4/Qocn)[, 2012)](https://paperpile.com/c/Hr0OH4/Qocn). The term T_k = 447.225 is a unitless arbitrary scaling factor.  

The Nuclear Pore Complex (NPC) model

Model components 

The simulated components include the NPC, the nuclear envelope (NE), the flexible FG repeat domains of FG Nups, and passively diffusing macromolecules, all enclosed within a bounding box. The configuration of these components is fully specified by a configuration vector  that includes all time-variable parameters, i.e. the spatial coordinates of the FG repeats and the passively-diffusing molecules. The system components were coarse-grained, similarly to their representation in Timney et al. 2016 to increase the computational efficiency of the simulations while reproducing key system properties regarding passive diffusion kinetics [(Timney ](https://paperpile.com/c/Hr0OH4/dZrI+90L9+MBad)[et al.](https://paperpile.com/c/Hr0OH4/dZrI+90L9+MBad)[, 2016; Kim ](https://paperpile.com/c/Hr0OH4/dZrI+90L9+MBad)[et al.](https://paperpile.com/c/Hr0OH4/dZrI+90L9+MBad)[, 2018; Raveh ](https://paperpile.com/c/Hr0OH4/dZrI+90L9+MBad)[et al.](https://paperpile.com/c/Hr0OH4/dZrI+90L9+MBad)[, 2024)](https://paperpile.com/c/Hr0OH4/dZrI+90L9+MBad)

Equilibration. 

All the diffusing molecules were initialized randomly at the upper side of the NPC. The model was then equilibrated for 1 microsecond with a step size of 2,000 fs at room temperature.  

| Component | Relevant IMP classes | Main parameters | Comments |
| --- | --- | --- | --- |
| Nuclear envelope (NE) + NPC scaffold | SlabWithCylindricalPore | NPC radius: 90 | The slab representing the NE is centered at Z=0 and oriented parallel to the XY plane. |
| FG repeats | Particle, XYZR, Diffusion | Bead radius: 8 nm Beads per chain (excluding anchor bead): 8 Diffusion coefficient: computed by Stokes’ law** Number of FGs per layer: 8 Number of layers: 1 or 3 (Results) | Each chain is represented as a flexible string of beads. An anchor bead was added as the last bead in each chain. Its center was fixed to the cylinder walls, in evenly spaced layers spanning the NE (from the cytoplasmic to nuclear end), and a radially symmetric pattern. |
| Passively-diffusing molecules | Particle, XYZR, Diffusion | Radius: 8-20 Å |  |
| Bounding Box | BoundingBox3D | Size: 130 x 130 x 130 Å3 or x 8 that for the 3 layer model |  |

* We use a downsized version of the real NPC [(Kim ](https://paperpile.com/c/Hr0OH4/90L9)[et al.](https://paperpile.com/c/Hr0OH4/90L9)[, 2018)](https://paperpile.com/c/Hr0OH4/90L9), similar to the one used in Timney et al. 2016, which was nonetheless shown to reproduce key system properties regarding passive diffusion [(Timney ](https://paperpile.com/c/Hr0OH4/dZrI+MBad)[et al.](https://paperpile.com/c/Hr0OH4/dZrI+MBad)[, 2016; Raveh ](https://paperpile.com/c/Hr0OH4/dZrI+MBad)[et al.](https://paperpile.com/c/Hr0OH4/dZrI+MBad)[, 2024)](https://paperpile.com/c/Hr0OH4/dZrI+MBad).
** using the IMP default based on the radius in IMP.atom.Diffusion

Table 4. Summary of the simple NPC model components, including technical representation in the Integrative Modeling Platform (IMP)[(Russel ](https://paperpile.com/c/Hr0OH4/Qocn)[et al.](https://paperpile.com/c/Hr0OH4/Qocn)[, 2012)](https://paperpile.com/c/Hr0OH4/Qocn).  

| Forces | Relevant IMP classes | Main parameters | Comments | Restrained particles |
| --- | --- | --- | --- | --- |
| Uexcluded | IMP.core. Excluded Volume Restraint | Spring constant    K Slack name | - | diffusers and FG |
| FG-bond | IMP.core. HarmonicDistancePairScore | center  Spring constant   k | - | All FG beads |
| non-specific | IMP.core. SphereDistancePairScore(IMP.core.TruncatedHarmonicLowerBound | center, Spring constant   k, threshold | - | diffusers and FGs |
| excluded-nuclea- envelope (NE) | IMP.npctransport. SlabWithCylindricalPore. setup_particle IMP.npctransport.SlabWithCylindricalPorePairScore IMP.core.PairRestraint | p_slab, slab_height, slab_pore_radius force constant k | - | diffusers |
| excluded-bounding-box | IMP.core.BoundingBox3DSingletonScore [IMP.core. HarmonicUpper Bound](https://integrativemodeling.org/2.19.0/doc/ref/classIMP_1_1core_1_1HarmonicUpperBound.html) IMP.container.SingletonsRestraint | IMP.algebra.BoundingBox3D distance, K_BB bb_harmonic,                                             bb | Constructed by IMP. algebra. Vector3D | all |

Table 5. Summary of interactions used in the NPC model, including technical representation in the Integrative Modeling Platform (IMP) [(Russel ](https://paperpile.com/c/Hr0OH4/Qocn)[et al.](https://paperpile.com/c/Hr0OH4/Qocn)[, 2012)](https://paperpile.com/c/Hr0OH4/Qocn).

| Component | Description/Class Used | Constants/Parameters | Values Extracted |
| --- | --- | --- | --- |
| Diffusers and FG | IMP.core.ExcludedVolumeRestraint | k_excluded, slack | k_excluded=1, slack=1 |
| Beads of FG repeats | IMP.core.HarmonicDistancePairScore | center, Spring constant (k) | center=distance_between_particles, k=2 |
| UFG-bond | IMP.core.HarmonicDistancePairScore | center, Spring constant (k) | center=16*1.9, k=2 |
| Diffusers and FG | IMP.npctransport.SlabWithCylindricalPore | slab_height, slab_pore_radius, k_slab | slab_height=55 or 110 for the finer model, slab_pore_radius=90, k_slab=5 |
| Uexcluded-bounding-box | IMP.core.BoundingBox3DSingletonScore | The bounding box (bb), distance, K_BB | bb=IMP.algebra.BoundingBox3D, K_BB=2 |
| All | IMP.core.SphereDistancePairScore | center, Spring constant (k), threshold | center=3, k=0.002, threshold=5 |

Table 6. Restraint parameters for the NPC model. including technical representation of these restraints in the Integrative Modeling Platform (IMP) [(Russel ](https://paperpile.com/c/Hr0OH4/Qocn)[et al.](https://paperpile.com/c/Hr0OH4/Qocn)[, 2012)](https://paperpile.com/c/Hr0OH4/Qocn).

Heuristic for choosing hyperparameters.

Selecting appropriate hyperparameters for TEMPO, particularly the simulation time step Δt (Algorithm 1) and recursion depth n, currently involves manual tuning. However, our experiments have empirically validated a practical heuristic that simplifies this process, enhancing its robustness and reliability.

In many practical settings, a suitable basic simulation time step for a naive simulation  is already known or can be reasonably guessed based on prior experience or standard guidelines within Brownian Dynamics (BD) methods. Even if a conservative estimate of   is not known in advance for a given system, it can be determined through methods like stability analyses over relatively short time scales, combined with testing stability and accuracy over longer time scales for a scaled-down version of the full system, rather than requiring exhaustive calibration for each new system.

Once an initial   is established, we propose, based on our empirical results,  that the TEMPO integrator's time step Δt in Algorithm 1 can be determined simply by multiplying   by 4 raised to the recursion depth (  × 4n). An important observation from our experiments is that recursive algorithms inherently tend to amplify errors at each recursion step compared to a gold-standard reference. Consequently, the recursion depth should be chosen to be as large as possible without compromising numerical stability. In practice, we observed that this optimal recursion depth can typically be identified by examining just a few initial steps of the TEMPO algorithm. Additionally, the chosen step size must be lower than the temporal resolution required by the measurement of interest. Based on our experience, this combined approach reliably yields near-optimal results. 

Applying this heuristic to simple systems such as the five and ten-bead models (Figures 3-4), we empirically determined that a  of approximately 0.24-0.28 fs yields minimal errors, comparable to smaller steps.  In these scenarios, a recursion depth of 5 is numerically stable, yielding the optimal Δt for TEMPO by multiplying   by 4 raised to the power of 5, whereas a recursion depth of 6 surpasses the relevant kinetic timescale, as indicated by the autocorrelation half-life, which is on the order of picoseconds (Figure 3D, 4B).

For more complex systems, such as simulations of nucleocytoplasmic transport through the nuclear pore complex (NPC), empirical hyperparameter tuning becomes somewhat more intricate. However, even in these cases, the time step can be obtained straightforwardly by calibrating on a scaled-down version of the full NPC [(Timney ](https://paperpile.com/c/Hr0OH4/dZrI+MBad)[et al.](https://paperpile.com/c/Hr0OH4/dZrI+MBad)[, 2016; Raveh ](https://paperpile.com/c/Hr0OH4/dZrI+MBad)[et al.](https://paperpile.com/c/Hr0OH4/dZrI+MBad)[, 2024)](https://paperpile.com/c/Hr0OH4/dZrI+MBad). In our experiments, we evaluated the naive algorithm performance against gold-standard simulations and empirically identified viable basic steps of 5,000 fs to 6,000 fs, while larger steps (e.g., 7,000 fs) exhibited substantial deviations from reference data (Figure 5A). Regarding recursion depth, empirical observations indicated numerical instability arising rapidly at depths exceeding 3, making recursion depth 3 the optimal empirically validated choice for NPC simulations.

Future work would automate this hyperparameter optimization further, streamlining the setup for new systems. This heuristic thus offers an empirically supported yet practical framework, balancing efficiency, accuracy, and ease of use across diverse applications.

Supplementary References.

[Kim,S.J. ](http://paperpile.com/b/Hr0OH4/90L9)[et al.](http://paperpile.com/b/Hr0OH4/90L9)[ (2018) Integrative structure and functional anatomy of a nuclear pore complex. ](http://paperpile.com/b/Hr0OH4/90L9)[Nature](http://paperpile.com/b/Hr0OH4/90L9)[, ](http://paperpile.com/b/Hr0OH4/90L9)[555](http://paperpile.com/b/Hr0OH4/90L9)[, 475–482.](http://paperpile.com/b/Hr0OH4/90L9)

[Raveh,B. ](http://paperpile.com/b/Hr0OH4/MBad)[et al.](http://paperpile.com/b/Hr0OH4/MBad)[ (2024) Integrative spatiotemporal map of nucleocytoplasmic transport. ](http://paperpile.com/b/Hr0OH4/MBad)[bioRxiv](http://paperpile.com/b/Hr0OH4/MBad)[.](http://paperpile.com/b/Hr0OH4/MBad)

[Rout,M.P. and Sali,A. (2019) Principles for Integrative Structural Biology Studies. ](http://paperpile.com/b/Hr0OH4/fiIwj)[Cell](http://paperpile.com/b/Hr0OH4/fiIwj)[, ](http://paperpile.com/b/Hr0OH4/fiIwj)[177](http://paperpile.com/b/Hr0OH4/fiIwj)[, 1384–1403.](http://paperpile.com/b/Hr0OH4/fiIwj)

[Russel,D. ](http://paperpile.com/b/Hr0OH4/Qocn)[et al.](http://paperpile.com/b/Hr0OH4/Qocn)[ (2012) Putting the pieces together: integrative modeling platform software for structure determination of macromolecular assemblies. ](http://paperpile.com/b/Hr0OH4/Qocn)[PLoS Biol.](http://paperpile.com/b/Hr0OH4/Qocn)[, ](http://paperpile.com/b/Hr0OH4/Qocn)[10](http://paperpile.com/b/Hr0OH4/Qocn)[, e1001244.](http://paperpile.com/b/Hr0OH4/Qocn)

[Sali,A. (2021) From integrative structural biology to cell biology. ](http://paperpile.com/b/Hr0OH4/oWaGq)[Journal of Biological Chemistry](http://paperpile.com/b/Hr0OH4/oWaGq)[, 100743.](http://paperpile.com/b/Hr0OH4/oWaGq)

[Timney,B.L. ](http://paperpile.com/b/Hr0OH4/dZrI)[et al.](http://paperpile.com/b/Hr0OH4/dZrI)[ (2016) Simple rules for passive diffusion through the nuclear pore complex. ](http://paperpile.com/b/Hr0OH4/dZrI)[J. Cell Biol.](http://paperpile.com/b/Hr0OH4/dZrI)[, ](http://paperpile.com/b/Hr0OH4/dZrI)[215](http://paperpile.com/b/Hr0OH4/dZrI)[, 57–76.](http://paperpile.com/b/Hr0OH4/dZrI)
